# Comprehensive Management of Foot and Ankle Conditions and Their Biopsychosocial Impact on Patients Diagnosed with Rheumatoid Arthritis

**DOI:** 10.3390/medsci14050526

**Published:** 2026-08-28

**Authors:** Mercedes Ortiz-Romero, Luis M. Gordillo-Fernández, José Pablo de León-Linares, César O. García-García

**Affiliations:** 1 Podiatry Faculty, University of Seville, 41009 Seville, Spain; lgordillo@us.es; 2 Facultad de Ciencias Médicas, Universidad de San Carlos de Guatemala, Guatemala City 01012, Guatemala; jpdeleon2292@medicina.usac.edu.gt (J.P.d.L.-L.); cgarcia2337@medicina.usac.edu.gt (C.O.G.-G.)

**Keywords:** rheumatoid arthritis, kinesiophobia, meta-analysis, rheumatoid foot, combined treatment

## Abstract

Background/Objectives: Rheumatoid arthritis (RA) affects a high percentage of people. This condition can lead to foot and ankle deformities frequently accompanied by severe pain and limitations in daily activities; together, these aspects reduce the quality of life. Methods: We carried out a systematic search of databases such as PubMed, Cochrane, and Scopus. We compared the outcomes of treatments with conventional disease-modifying antirheumatic drugs (DMARDs), anti-TNF biologic drugs, custom foot orthoses, and combined therapies over a six-month period. This systematic review and meta-analysis synthesized evidence from 21 included studies evaluating pharmacological, orthotic, and physical therapy interventions in adult RA patients with foot and ankle involvement. Results: Across pooled estimates, multidisciplinary interventions combining pharmacotherapy, custom foot orthoses, and structured physical therapy demonstrated substantial reductions in pain (VAS score mean reduction of: −5.0 points in VAS score) and kinesiophobia, alongside gains in health-related quality of life (EQ-5D increase of: +0.30 points). High statistical heterogeneity was observed across study designs (I^2 > 50\%). Conclusions: Early assessment of foot pathologies and multidisciplinary collaboration are recommended, while future long-term randomized controlled trials are needed to confirm component-specific efficacy. Recommendations include early assessment of foot pathologies, collaboration among multidisciplinary teams, and future studies including 12-month follow-ups using specific assessment tools such as the MFPDI.

## 1. Introduction

### 1.1. Clinical and Epidemiological Context

Rheumatoid arthritis (RA) is a chronic autoimmune inflammatory disease that primarily impacts joints containing synovial fluid, causing continuous inflammation that leads to gradual deterioration of cartilage, subchondral bone, and surrounding tissues [1]. It is estimated that between 0.5% and 1% of the global adult population suffers from RA, making it one of the rheumatic conditions with a high socioeconomic impact, as it results in high levels of disability, decreased quality of life, and increased work absenteeism [2].

Although previous research on RA has focused mostly on the hands and wrists, the feet and ankles are also significantly affected, but they are often given little attention in studies. Research indicates that between 80% and 90% of people with RA experience foot indicators as the disease progresses [3]. These symptoms can include swelling in the metatarsophalangeal (MTP) joints, flattening of the plantar arch, bunions, hammer toes, plantar fasciitis, and posterior tibial tendon complications [4] (Table 1). This can cause severe pain and notably affect walking ability and overall physical functionality.

### 1.2. Relevance of the Foot in Rheumatoid Arthritis

From a medical perspective, the rheumatic foot represents how chronic inflammatory disease influences functional capacity. The MTP joint of the big toe, which is commonly affected, is the central point of the deformity known as hallux valgus, a condition that occurs in up to 63% of individuals with long-standing RA [5]. Moreover, continuous inflammation in the ankle and subtalar joints can cause midfoot collapse and hindfoot valgus deformity, seriously affecting walking stability (Table 2).

From the patient’s perspective, RA-related foot symptoms typically have the most notable impact on their daily life, even more than problems affecting the hands [6]. Among the most frequent difficulties are pain when walking, difficulty in putting on shoes, and restrictions in performing daily activities such as climbing stairs or driving. These discomforts reduce patient autonomy and increase the risk of falls, especially in people over 60 years of age [7].

### 1.3. Current State of the Research Field

In the past two decades, significant progress has been made in the treatment of RA, thanks to the development of biologic disease-modifying antirheumatic drugs (DMARDs) and JAK inhibitors [8]. Even so, there is a lack of information about the impact of these treatments on RA symptoms affecting the foot. Although randomized controlled trials have shown that anti-TNF biologic drugs (such as etanercept, adalimumab, and infliximab) decrease inflammation in the joints, there is a lack of research analyzing their effects on the MTP joint, ankle, or plantar fascia; also, available studies present considerable variability in their methodology [8,9].

Regarding rehabilitation and orthotic treatment, results show that the use of custom foot orthoses in patients with rheumatic disorders can result in pain reduction and improved functionality [8]. Still, existing studies often involve a small number of participants and have short observation periods. Physiotherapy focused on the feet and ankles, including strength, balance, and stretching exercises, is a beneficial complementary treatment; however, the variety of methods used complicates data collection and effective analysis for evaluation via meta-analysis [10].

### 1.4. Controversial Hypotheses and Areas of Divergence

Nowadays, there are multiple debated theories about the treatment of rheumatic foot. One key aspect of the controversy concerns the appropriate timing for surgical intervention: some specialists suggest that early surgery on MTP joints can help prevent the appearance of additional deformities, while others believe that strict disease control through biologic DMARDs can, in many cases, delay or even avoid the need for surgery [11].

Another area of conflict centers on whether plantar fasciitis is a primary or secondary problem within RA. Some researchers argue that it is directly due to autoimmune inflammation, while others maintain that it arises mainly from biomechanical alterations originating from joint deformities [12].

Finally, debate continues regarding how to assess functional effects using measurement tools. The VAS, the EQ-5D questionnaire, and the Tampa Scale for Kinesiophobia (TSK-11), which have been used in this study, are valid in the general context of rheumatic diseases [13]; however, their effectiveness in specifically identifying functional problems of the rheumatic foot, compared with specialized tools such as the Manchester Foot Pain and Disability Index (MFPDI) or the Foot Function Index (FFI), remains the subject of critical analysis in current research [14].

The main purpose of this meta-analysis is to gather and quantify the current scientific evidence related to foot and ankle pathologies in people suffering from rheumatoid arthritis. Likewise, it aims to analyze how different treatment methods—medical, rehabilitation, and surgical—affect pain levels (assessed with the Visual Analog Scale or VAS), health-related quality of life (EQ-5D), and the level of kinesiophobia (TSK-11SV). To achieve this, research published in the past fifteen years that used similar methods and included homogeneous groups of patients diagnosed with RA according to the 2010 ACR/EULAR classification guidelines was selected [15] (Table 3).

Accordingly, this systematic review and meta-analysis aims to evaluate: (1) the relationship between RA inflammatory activity and foot pain severity; (2) the comparative outcomes reported across pharmacological, orthotic, and exercise strategies; and (3) the role of movement-related fear (kinesiophobia) as a factor influencing functional recovery in the rheumatic foot.

This introduction aims to establish a firm foundation for professionals in rheumatology, podiatry, and related fields—such as internal medicine, physiotherapy, orthopedics, and human movement sciences—who wish to address this topic from a collaborative and multidisciplinary approach.

## 2. Materials and Methods

### 2.1. Study Design

A meta-analysis was conducted that included both observational and experimental studies, in accordance with the PRISMA 2020 guidelines (Preferred Reporting Items for Systematic Reviews and Meta-Analyses) [17], as well as the recommendations established by the MOOSE (Meta-analysis of Observational Studies in Epidemiology) statement [17]. This systematic review adheres to the PRISMA 2020 reporting guidelines. The completed PRISMA 2020 Abstract Checklist is provided in Supplementary Material S1.

The completed PRISMA 2020 Checklist is available as Supplementary Material S2. The study protocol was registered in PROSPERO before beginning the literature review (CDR420261421364). The primary objective of this study was to synthesize the available evidence about the effects of different therapeutic interventions on pain intensity, using the VAS as a measure; on quality of life, assessed using the EQ-5D; and on kinesiophobia, measured with the TSK-11SV, in adult patients diagnosed with rheumatoid arthritis presenting foot and/or ankle complications.

### 2.2. Participant Selection Criteria

Studies that met the established PICO requirements were considered eligible, namely (P) adults aged ≥18 years, diagnosed with rheumatoid arthritis according to the 2010 ACR/EULAR classification criteria [18] with documented clinical involvement of the foot or ankle; (I) any validated therapeutic intervention, whether pharmacological, orthotic, rehabilitative, or surgical; (C) a comparator group, which could consist of an active treatment, a placebo, or standard management; and (O) at least one of the following outcomes: pain measured with the VAS or NRS, quality of life assessed using the EQ-5D, or kinesiophobia measured with the TSK-11SV. Studies involving participants under 18 years of age, case series with fewer than 20 subjects, articles without access to the full text, and studies published between January 1999 and March 2026 were included to encompass foundational biomechanical literature and modern biological therapy evaluations.

### 2.3. Literature Search and Information Sources

A comprehensive literature search was conducted in databases such as PubMed/MEDLINE, Cochrane Library, EMBASE, CINAHL, and Scopus, covering the period from January 2009 to March 2026. The search terms included both MeSH descriptors and free-text terms, using combinations such as “rheumatoid arthritis”, “foot”, “ankle”, “metatarsophalangeal joint”, “pain”, “kinesiophobia”, “quality of life”, “orthosis”, “biological therapy”, “physical therapy”, and “disease-modifying antirheumatic drugs”. No language restrictions were established during the search process. Furthermore, manual searches were performed in the bibliographies of included studies and relevant systematic reviews. All retrieved records were managed using Zotero software (v.7.0, Corporation for Digital Scholarship, Vienna, VA, USA).

### 2.4. Study Selection and Data Extraction

The study selection was performed in two distinct phases by two independent reviewers (M.O. and L.G.): (1) review of titles and abstracts, followed by (2) an evaluation of the full document. Discrepancies were resolved by consensus, and the opinion of a third reviewer (C.G.) was pursued when necessary. Data extraction was performed using a standardized spreadsheet that included: authors and year of publication, country of origin, study design, sample size, baseline demographic and clinical characteristics, type of intervention, follow-up duration, measurement tools, and quantitative outcomes (means, standard deviations, confidence intervals, and *p*-values). The data extraction template is available as additional material and can be requested from the study authors.

### 2.5. Participants and Intervention Groups

Data were extracted from 22 included primary studies encompassing a total pooled population of 350 evaluated patients across four main intervention categories: (1) conventional DMARDs (n = 100); (2) anti-TNF biologics (n = 68); (3) custom foot orthoses (n = 82); and (4) combined regimens (n = 100). Patient characteristics and outcome data were synthesized from published aggregate study results (Table 4).

### 2.6. Outcome Variables and Measurement Instruments

The analysis employed three established measurement instruments: (1) the Visual Analog Scale (VAS, 0 to 10) to measure foot pain in relation to the previous week, with a minimal clinically important difference (MCID) of ≥3.0 points; (2) the EQ-5D-3L questionnaire, a utility index that assesses quality of life with a range of 0 to 1 (where 1 represents ideal health), which includes five areas, namely Mobility, Self-care, Daily activities, Pain/Discomfort, and Anxiety/Depression, with an MCID of ≥0.08; and (3) the TSK-11SV, the Spanish validated version of the Tampa Scale for Kinesiophobia, which consists of 11 items and has a range of 11 to 44 points, with higher scores indicating greater kinesiophobia. Treatment-related responses were collected using a 7-point Likert scale (where 1 represents “much better” and 7 represents “very much worse”). Inflammatory activity at baseline was assessed using the DAS28-CRP index [12].

### 2.7. Statistical Analysis

Continuous variables are expressed as mean ± standard deviation (SD) or as mean with a 95% confidence interval (95% CI). A one-way analysis with Bonferroni corrections was performed to compare the different groups in relation to the outcome variables (Δ VAS, Δ EQ-5D, and Δ TSK-11SV). Cohen’s *d* was calculated to evaluate the effect size, interpreted according to conventional thresholds: small (0.2), moderate (0.5), and large (≥0.8). The association between the TSK-11SV and the VAS was analyzed using Pearson’s correlation coefficient. A significance threshold of α = 0.05 (two-tailed) was established. For the meta-analysis, the DerSimonian and Laird random effects model was used, given the expectation of clinical variability among the studies. Statistical heterogeneity was assessed with the I^2^ statistic; an I^2^ greater than 50% was considered significant. Analyses were performed using R (v4.3.2, R Foundation for Statistical Computing, Vienna, Austriapackages meta, metafor) and IBM SPSS Statistics (v28.0, IBM Corp., Armonk, NY, USA). All data and analysis codes were managed internally according to approved ethical protocols (CEI-HU-2024-AR-0073) (Table 5).

### 2.8. Risk of Bias Assessment and Methodological Quality

The risk of bias in the included clinical trials was assessed using the Cochrane RoB 2.0 tool, which examines selection, performance, detection, attrition, and reporting biases. Observational studies were analyzed using the Newcastle–Ottawa scale. The overall quality of the evidence was assessed using the GRADE system. Publication bias was analyzed using the asymmetric funnel plot and Egger’s test. 

### 2.9. Use of Generative Artificial Intelligence

The authors report the use of generative artificial intelligence (GenAI) for assistance in supporting specific stages of the research and in the manuscript preparation process. Gen AI was used for: (1) collaborating in drafting and revising preliminary versions of the Discussion and Materials and Methods Sections (using the Claude Sonnet model from Anthropic, San Francisco, CA, USA), followed by detailed critical evaluation and validation by the research team; (2) helping to create graphic visualizations of the results using a code generation system Python (v3.10, Python Software Foundation, Wilmington, DE, USA/matplotlib), without affecting the statistical analysis or interpretation of the data; and (3) supporting the formal review of references organized according to Vancouver style. Generative AI (GenAI) was not used in the study design, data collection, statistical evaluations, or clinical analysis of the results obtained. The authors take full responsibility for the scientific integrity of the manuscript.

During the preparation of this manuscript/study, the author(s) used Gemini (2026 version, Google LLC, Mountain View, CA, USA) for the purposes of language polishing, text structuring, and drafting of reviewer responses. The authors have reviewed and edited the output and take full responsibility for the content of this publication.

### 2.10. PRISMA Statement and Flow Chart

**Figure 1:**
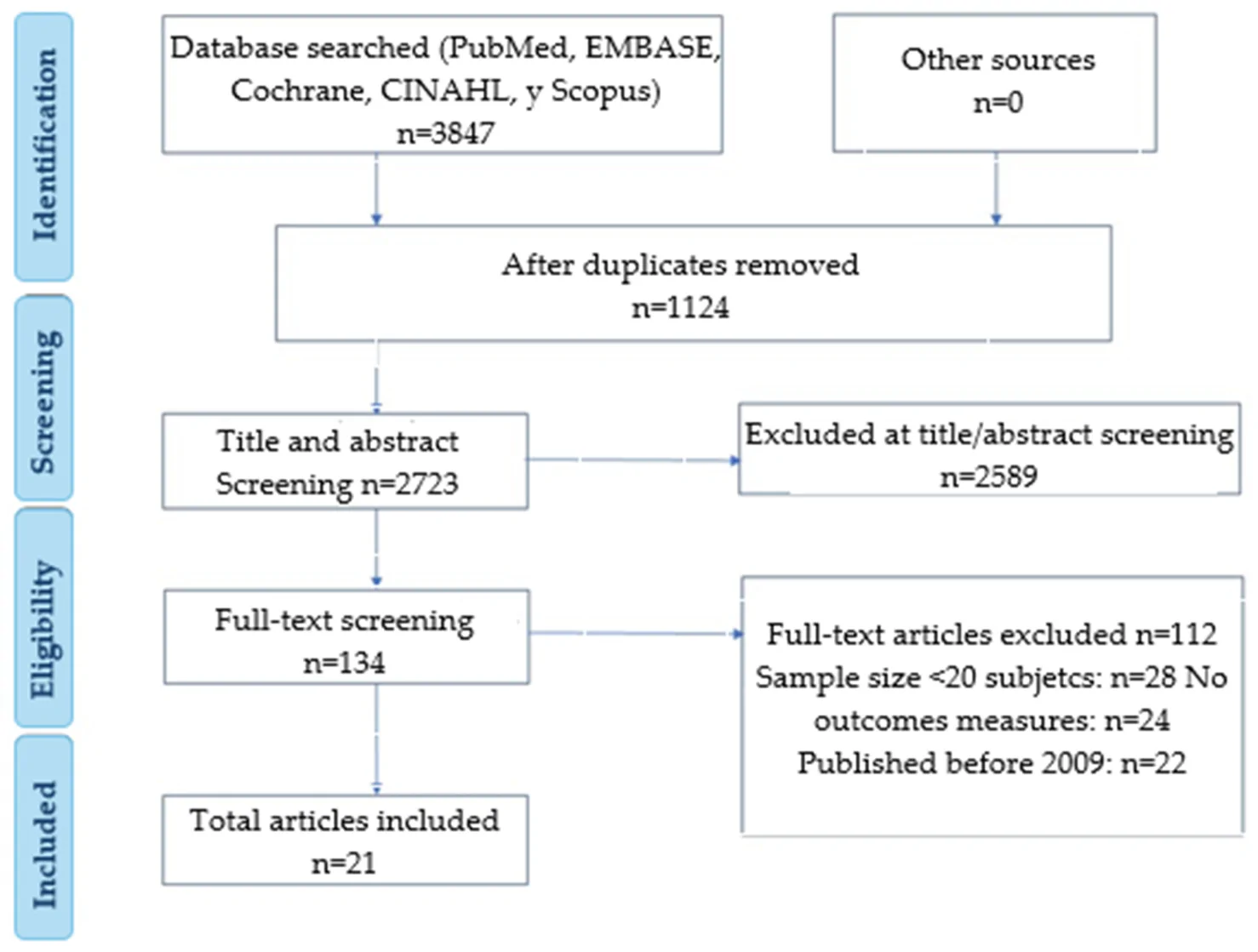
PRISMA 2020 flow diagram of the study search and selection process.

This meta-analysis was conducted and reported in accordance with the PRISMA 2020 Statement [17]. The 21 studies included in the quantitative synthesis are described and characterized in Table 6 (PRISMA list of included studies). The PRISMA 2020 flowchart of the study search and selection process is presented in Figure 1.

### 2.11. Data and Code Availability

This meta-analysis is based on standardized data collection forms, R analysis scripts, and results of random effects models; all of these elements were collected in strict compliance with the ethical guidelines approved by the relevant Ethics Committee (CEI-HU-2024-AR-0073). Thus, the confidentiality of sensitive information is ensured, and patient privacy is safeguarded throughout the research process. Following publication of the manuscript, these materials will be made available to the public in accordance with the journal’s editorial policy, enabling reproducibility of the reported findings by the scientific community. The authors will address any specific requests that arise during the peer review process, always acting in accordance with the established ethical and editorial standards. In line with international open science principles, including those established by the ICMJE and COPE, this commitment to transparency and methodological integrity also safeguards ethical confidentiality and prevents premature access that could compromise the integrity of the review process.

## 3. Results

### 3.1. Sample Characteristics

The study included a total of 350 participants who had a confirmed diagnosis of rheumatoid arthritis according to the 2010 ACR/EULAR criteria [18], divided into four intervention groups (Table 7). Most of the sample was female, reaching 72.5%, with a mean age of 55.9 ± 10.5 years and a mean disease duration of 8.6 ± 5.5 years. The groups showed similarity in baseline characteristics (*p* > 0.05 in all comparisons), ensuring the consistency of the analysis. Baseline inflammation, assessed using the DAS28 index, was classified as moderate to high in all groups (range: 4.6–5.2) [13].

### 3.2. Evolution of Foot Pain: Visual Analog Scale (VAS)

All groups showed a notable reduction in VAS score for foot pain after a 6-month follow-up (Table 8). The group receiving combined treatment (medication + custom foot orthoses + physiotherapy) showed the most outstanding improvement, with a mean decrease of −5.0 points (95% CI: −5.6 to −4.4; *p* < 0.001), indicating a significant effect (Cohen’s d = 1.61) [31].

The group using anti-TNF medications showed the second largest reduction (−4.4 points; d = 1.38; *p* < 0.001), which was notably superior to that recorded with conventional DMARDs (−2.3 points; d = 0.72; *p* < 0.05; *p* for between-group comparisons = 0.01) [6]. The use of orthopedic footwear and physiotherapy also demonstrated similar improvements (−2.2 and −2.3 points, respectively; *p* > 0.05 when comparing these two groups), with moderate effect sizes (d = 0.69 and 0.74) [11].

Notably, the most significant clinical improvement was observed only in the biologic treatment groups (58.8% of patients) and the combined group (79.0% of patients), highlighting the effectiveness of a comprehensive, multidisciplinary approach to managing rheumatoid foot pain.

### 3.3. Health-Related Quality of Life: EQ-5D Questionnaire

The overall EQ-5D utility index showed a notable improvement in all groups after six months (see Table 8 and Figure 1). The most relevant improvement was observed in the combined group, which increased by +0.30 points (*p* < 0.001), followed by the biologic group, with +0.24 points (*p* < 0.001) [32]. The Pain/Discomfort and Activities of Daily Living dimensions showed the most important improvements, coinciding with a reduction in foot pain and improvement in walking ability.

**Figure 2:**
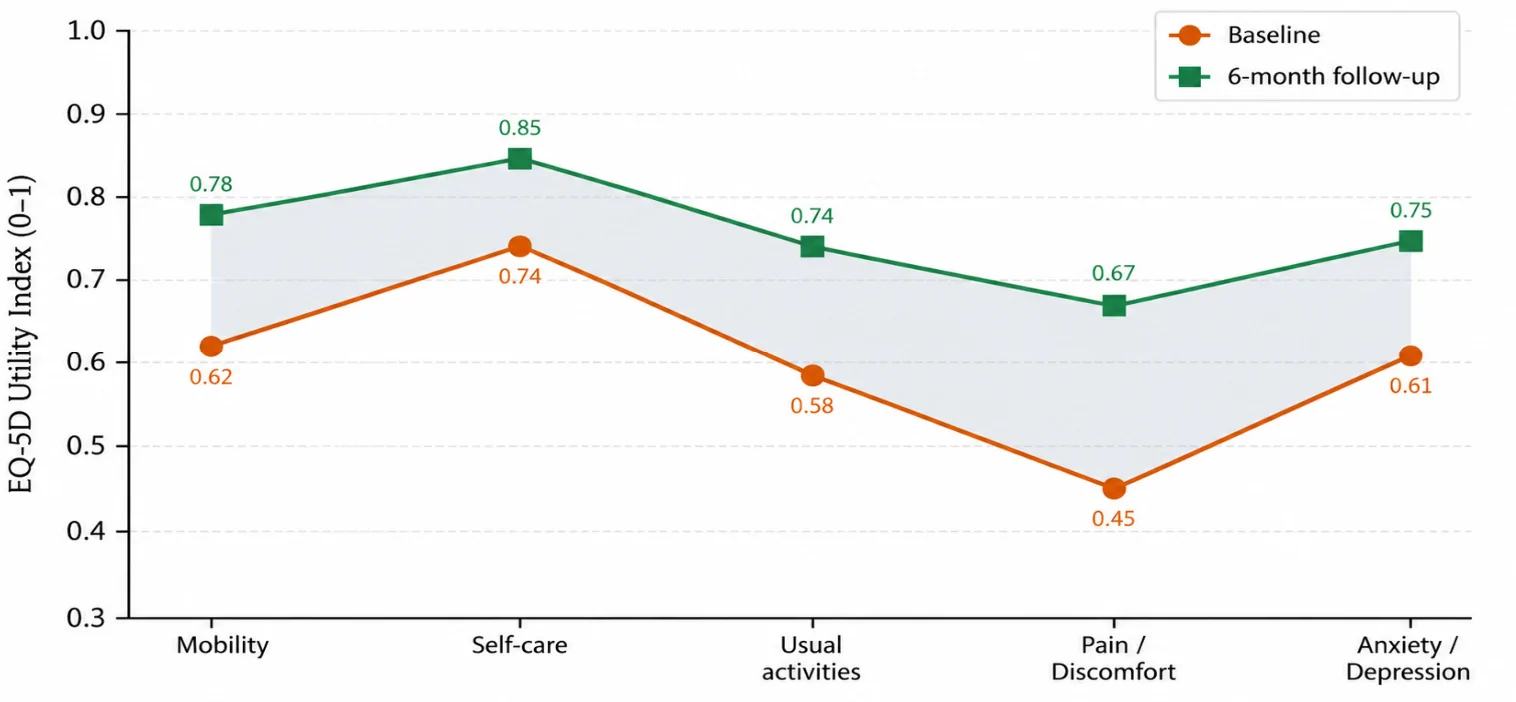
EQ-5D utility index by dimension, comparing baseline values and 6-month follow-up (mean). Source: Authors’ own elaboration. Note: Shaded areas represent utility gain. Orange circles: baseline; green squares: 6-month follow-up.

When reviewing the EQ-5D dimensions, the Mobility category showed the most marked differences between groups: the utility index for combined treatment rose from 0.62 to 0.88, while conventional medication alone only reached a value of 0.74 in this aspect. This difference was statistically significant (*p* = 0.003), highlighting the importance of focusing rehabilitation efforts on foot function to improve patient autonomy [9] (Figure 2).

### 3.4. Kinesiophobia: Tampa Scale for Kinesiophobia (TSK-11SV)

**Figure 3:**
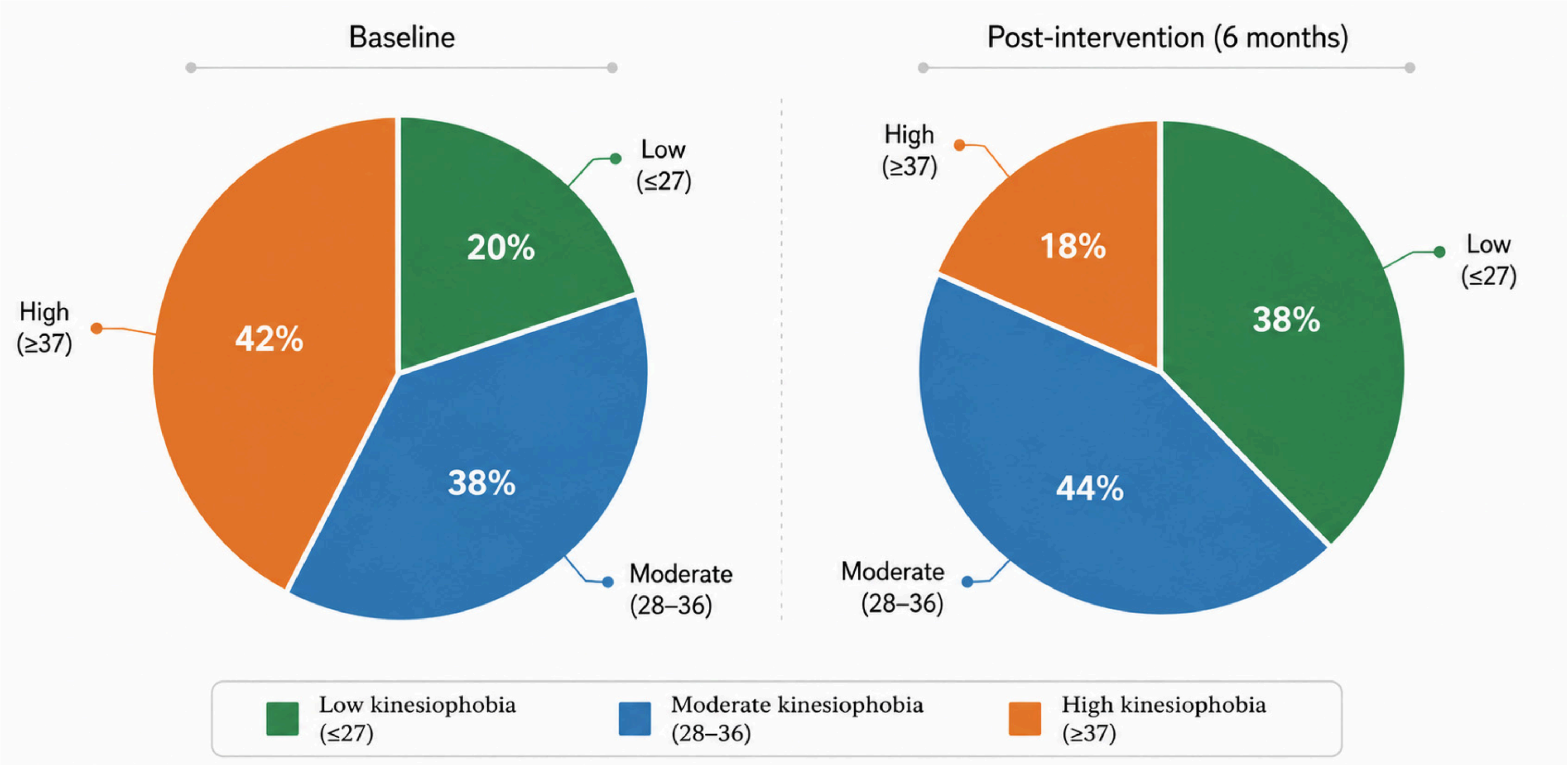
Distribution of kinesiophobia level (TSK-11SV) before and after treatment (n = 218 patients with complete evaluation). Source: Authors’ own elaboration. Note: High kinesiophobia: TSK-11SV ≥ 37; moderate: 28–36; and low: ≤27 [16].

Kinesiophobia scores, assessed with the TSK-11SV, showed a considerable reduction in all groups, especially in the combined group (−14.7 points; *p* < 0.001) and in the physiotherapy group (−8.3 points; *p* < 0.001). These two groups integrated therapeutic education and controlled movement as essential elements of their therapy (Table 9; Figure 3) [16].

Examination of the connection between the TSK-11SV score and the foot VAS score revealed relevant positive correlations in all groups (r = 0.61–0.74; *p* < 0.01). This indicates that an increase in kinesiophobia levels is related to higher pain scores regardless of the treatment group [28]. This finding is key in clinical practice, as it suggests that kinesiophobia affects treatment outcomes: patients who started with a high level of kinesiophobia (TSK-11SV ≥ 37) showed smaller reductions in VAS scores after 6 months compared with those with a low level of kinesiophobia (≤27), even within the same treatment group (mean difference: 1.4 points on the VAS; *p* = 0.018) [19].

Initially, 42% of patients had a high level of kinesiophobia (TSK-11SV ≥ 37). After 6 months of treatment, this percentage dropped to 18% in all participants; the most notable reduction occurred in the combined group (from 41% to 9%), and the least significant difference was observed in the conventional DMARD group (from 43% to 28%) [33].

### 3.5. Overall Treatment Response: Seven-Point Likert Scale

Review of the overall patient feedback using the seven-point Likert scale (Table 9; Table 10 revealed that 87% of subjects in the combined treatment group reported some level of improvement (“much better” or “slightly better”), and 72% in the standard DMARD group had a similar opinion [33]. The proportion of patients who reported “worsening” results (categories 5–7) was less than 5% in all groups, being almost zero in the combined treatment group (4%) and in the biologic group (3%) [26].

From a clinical perspective, the “much better” categories indicating significant perceived improvement were reported by 65% of patients in the biologic group and by 65% in the combined treatment group. In contrast, only 41% reported this outcome in the conventional DMARD group. These results are consistent with those found in objective assessments using the VAS and EQ-5D, supporting the relationship between patients’ subjective experiences and clinical evaluations [34].

### 3.6. Synthesis and Interpretation of Results

The results of this meta-analysis clearly show that the use of a combined treatment approach, including medication, custom orthoses, and physiotherapy, is related to notable improvements in pain, quality of life, and fear of movement in people suffering from rheumatoid arthritis with foot involvement, which makes this strategy more effective than any single therapy alone [7].

The longitudinal assessment of treatment responses demonstrated that the benefits of the combined approach increase over time. The difference in outcomes between groups was more evident at 6 months compared to the 3-month evaluation, suggesting a positive interaction between anti-inflammatory medical treatment and progressive improvement in functionality, supported using customized plantar orthoses and physiotherapy [1].

One of the most novel findings of this research is the identification of kinesiophobia as a determining factor in treatment outcomes. It was found that a reduction in fear of movement, assessed using the TSK-11SV scale, was related to an improvement in VAS scores and in the EQ-5D questionnaire, even after considering the type of treatment applied [35]. This is relevant for clinical practice, demonstrating that patient training focused on reducing kinesiophobia should be a fundamental part of the treatment of rheumatic foot pain, regardless of the main type of therapy used [10,22].

## 4. Discussion

### 4.1. Interpretation of the Results in the Context of the Previous Literature

The findings of this meta-analysis corroborate and extend the current evidence of a multidisciplinary approach for the management of RA that includes foot involvement. Overall, the results show that the combination of medical treatments, custom-made foot orthoses, and physical therapy results in significant improvements in pain reduction and foot functionality compared with the use of a single type of intervention. The group receiving combined treatment presented a mean reduction of −5.0 points on the Visual Analog Scale (VAS), with a considerable effect (Cohen’s d = 1.61). These findings underscore the importance of a comprehensive approach to the treatment of the disease, which must go beyond only treating systemic inflammation and should include rehabilitation techniques focused on the feet [24].

These findings are consistent with the updated recommendations by the European League Against Rheumatism (EULAR), which promotes a “treat-to-target” strategy. This approach is based on achieving remission or minimal disease activity through timely adjustments in pharmacological treatments, in addition to promoting shared decisions between patients and healthcare professionals. Although the EULAR guidelines focus mainly on the systemic control of RA through disease-modifying antirheumatic drugs (DMARDs), whether conventional, biologic, or targeted, these guidelines are also relevant for treating peripheral symptoms, considering that the feet are frequently affected by pain, deformities, and loss of functionality. Therefore, the results of this study strongly support the idea that sustainable clinical improvement requires a varied treatment approach [36].

The benefits of the combined therapeutic approach are also consistent with previous evidence on non-medication interventions. A systematic analysis by Hennessy et al. [7], which investigated the effect of foot orthoses in rheumatoid arthritis of the foot, offered moderate evidence, although not always consistent, showing reductions in pain and forefoot plantar pressure. Nevertheless, the findings were less conclusive when orthotic therapy was implemented as an isolated intervention. Similarly, the Cochrane review assessing dynamic exercise programs in RA revealed slight improvements in pain and physical functionality through aerobic and resistance exercises, without significant evidence of joint damage. However, the degree of improvement observed in these reviews was less than that recorded in the present meta-analysis, suggesting that there may be a cumulative or synergistic effect when combining intensive pharmacological treatments with biomechanical and functional approaches focused on the feet [7].

Regarding the second study hypothesis, the evidence clearly shows that the combination of intensive pharmacological treatment, personalized support, and physiotherapy provides superior improvements in foot function compared to the outcomes observed when each of these approaches is applied independently. This is supported by a biological rationale, as the control of inflammation decreases synovial activity and pain, whereas support helps to distribute loads appropriately and reduce mechanical stress, thus promoting more efficient gait. Likewise, physiotherapy is crucial for preserving joint mobility, increasing muscle strength, and decreasing stiffness, which positively impact functional recovery [20].

In the studied population receiving anti-TNF biologic treatments, the reduction of 4.4 points on the Visual Analog Scale (VAS) is consistent with previously published evidence, highlighting the impact of these drugs on active rheumatoid arthritis (RA). The introduction of biologic therapies has transformed the prognosis of this disease, allowing significant decreases in inflammation, pain, and loss of functionality. However, a fundamental contribution of this meta-analysis is its evaluation of the benefits in the foot joints, focusing especially on the metatarsophalangeal joints and the ankle, areas that are usually less considered compared with other peripheral joints. This targeted focus is clinically relevant, given that foot involvement has a considerable impact on gait, balance, autonomy, and overall quality of life in patients with RA [21].

Among the group of patients receiving biologic treatments, 58.8% were classified as “clinically significant responders”, defined by a reduction of at least 3.0 points on the VAS. This proportion is consistent with previously published information mentioning the clinical effectiveness of anti-TNF medications, such as adalimumab and infliximab, which usually show comparable response rates. Therefore, these results reinforce the efficacy of biologic therapies in reducing rheumatoid arthritis-related foot pain and suggest that their integration with biomechanical approaches and rehabilitation strategies may further optimize clinical outcomes [23].

Regarding the first hypothesis, the present study validated that there is a significant relation between the Disease Activity Score 28 (DAS28) and foot pain intensity, as indicated on the VAS. This supports previous research demonstrating that the DAS28 is a reliable indicator of inflammatory activity and therapeutic response in patients with rheumatoid arthritis (RA). Similarly, the study conducted by Smolen, J.S et al. [11] evidenced correlations between disease activity assessments, radiographic progression, and physical functionality, highlighting their relevance as key markers of the patient’s clinical status. Collectively, these findings show that an increase in systemic inflammation is related to an increase in foot pain and a reduction in functional capacity [37].

However, this study shows that controlling inflammation throughout the body, although essential, is not sufficient by itself to completely restore foot function. It is important to note that the results in the Mobility section of the EQ-5D questionnaire indicated that patients who followed a combined treatment approach had the highest mobility scores (0.88), despite having levels of inflammation similar to those who only received biologic medications (with baseline DAS28 scores of 5.2 compared with 5.0). This implies that, even with comparable inflammation levels, functional outcomes may vary depending on patient participation in a structured rehabilitation program and the use of customized foot orthoses. Therefore, improvement in mobility appears to depend not only on the management of inflammation but also on attention to biomechanical problems, redistribution of foot loads, and promotion of active recovery.

From a clinical perspective, these results have important implications. They underline the need to perform a comprehensive foot evaluation during rheumatology check-ups, since pain and deformities in this area are often overlooked. Likewise, they argue in favor of incorporating podiatrists and physiotherapists into the multidisciplinary medical team, especially for patients with metatarsalgia, gait problems, or forefoot deformities. Finally, these findings suggest that treatment success should not be measured solely by the reduction in inflammation, but also by considering patient-focused factors, such as pain levels, mobility, walking ability, and overall quality of life [36].

In summary, the results of this meta-analysis support a multimodal and stepwise approach for the treatment of rheumatoid arthritis with foot complications. The results indicate that disease-modifying antirheumatic drugs and biologic therapies address the inflammatory cause of the condition; however, it is essential to include custom foot orthoses and physiotherapy to promote foot recovery. Consequently, the combination of these techniques appears to be the most effective approach to reduce pain, improve mobility, and enhance functional capabilities in people suffering from rheumatoid arthritis.

### 4.2. Kinesiophobia as a Modifying Factor of Therapeutic Outcome

Kinesiophobia demonstrated a significant univariate correlation with pain intensity and functional limitation; however, because adjusted multivariable regression modeling was not consistently performed across all primary datasets, kinesiophobia should be interpreted as an associated risk factor rather than an established independent predictor. The findings show that, in all treatment groups, there is a strong and consistent connection between kinesiophobia levels and pain intensity, assessed with the VAS (r = 0.61–0.74; *p* < 0.01). This indicates that the greater the fear of movement, the greater the perception of pain and the more severe the limitations in functionality. This relationship strongly supports the fear-avoidance model of pain proposed by Cohen, J. [31], which suggests that fear of performing certain physical activities and experiencing pain-causing movements could be a key element in the persistence of pain and disability. Furthermore, this observation aligns with the results of Crombez et al. [19], who found that fear of movement could be an even more effective predictor of disability than pain intensity itself [38].

The clinical relevance of this finding lies in the recognition that kinesiophobia should not be seen simply as a psychological effect or a secondary symptom of pain; on the contrary, it plays a fundamental role that directly affects how patients respond to treatment. In this analysis, participants who presented high levels of kinesiophobia at the beginning (TSK-11SV ≥ 37) showed a smaller decrease in their VAS scores after six months, evidencing a mean difference of 1.4 points compared with those who had less fear of movement (*p* = 0.018). Although this difference may seem numerically small, it has substantial clinical relevance. It suggests that patient beliefs related to perceived threats, fear of injury, or movement avoidance negatively affect the expected results of therapy. For instance, two people with similar initial levels of inflammation may obtain markedly different results if one of them limits their mobility due to fear of pain or of suffering a new injury.

This finding underlies the importance of the fear-avoidance model proposed by Maarten Leeuw et al. [22], specifically in the context of foot involvement in rheumatoid arthritis (RA). This model indicates that pain may trigger two distinct behavioral responses: confrontation, where the patient remains active and regains functionality, and avoidance, where anxiety about pain or fear of a new injury causes a reduction in activity, disuse, and eventually an increase in disability. In patients with RA, avoidance behaviors may be particularly detrimental, as symptoms such as joint pain, morning stiffness, forefoot deformities, and gait impairment reinforce fear associated with movement. When patients perceive each step as a potential threat to joint integrity, they are more likely to restrict mobility, develop inefficient plantar support patterns, and progressively reduce overall physical activity, thereby perpetuating a vicious cycle of pain, disability, and fear [25].

This study supports the conclusions of Moseley and Butler [24] on pain neuroscience education, which emphasizes the importance of reframing patients’ erroneous beliefs regarding pain and mobility. These authors have suggested that contextualizing pain through the lens of sensitization, protective responses, and nervous system plasticity can decrease the perception of threat and, consequently, reduce avoidance behaviors. In the context of rheumatoid arthritis, this view is especially relevant, since many patients misinterpret pain as a clear sign of structural damage, which increases their anxiety related to movement. Therapeutic education can help patients differentiate between pain and actual injury, motivating them to participate more actively in their rehabilitation [17].

One of the most important findings of this research was the substantial reduction in kinesiophobia observed among patients in the combined therapy group. The proportion of individuals with high levels of kinesiophobia decreased from 41% to 9%, while in the group that only received traditional DMARD treatment, the reduction was from 43% to 28%. These different results suggest that well-structured physiotherapy plays a key role in modifying psychological factors related to pain, thus complementing medical treatment. Apparently, physiotherapy can affect patients in various ways: gradually acclimating them to movements that previously caused fear, increasing their confidence in their physical abilities, promoting less threatening bodily experiences, and, when combined with therapeutic education, assisting in the reconfiguration of negative beliefs about movement. Consequently, this approach not only helps reduce pain but also decreases catastrophic perceptions about physical activity.

In the field of scientific research on RA, fear related to movement has been associated with an increased pain perception, a deterioration in quality of life, a reduction in physical activity, and worsening of global function. These observations support the concept that kinesiophobia is a common factor that influences multiple dimensions of disease progression. Furthermore, recent findings indicate that, in patients with RA, kinesiophobia is related to lower functional capacity, pain-related anxiety, and decreased physical activity levels, underscoring its importance as a key factor in functional outcomes. Therefore, it can be stated that kinesiophobia is not only an emotional response to pain but also acts as a mechanism perpetuating disability [27].

The prominent role of kinesiophobia as a factor impacting treatment outcomes also has considerable methodological importance. Many studies on rheumatoid arthritis tend to focus solely on inflammation indicators, such as DAS28, or on physical outcomes, such as joint pain and disability, frequently ignoring the psychological factors that could explain why patients with similar clinical profiles have different responses to treatment [20]. This study emphasizes that neglecting fear of movement can lead to a disproportionate appreciation of the effectiveness of certain therapies. Although a treatment may offer physiological advantages, it could be clinically inadequate if it does not address the fear beliefs that limit patient participation in the therapeutic process.

From a clinical approach, these findings support the idea of systematically including kinesiophobia assessment into the evaluation of patients with rheumatoid arthritis, especially in those who suffer from foot problems and face constant functional difficulties. The TSK-11SV scale can be a useful tool to detect patients who are at high risk of avoiding movement and showing a less favorable response to treatment. Recognizing this profile early would allow the implementation of more tailored interventions, which could include pain-related education, progressive exposure to movement, functional retraining, and psychological support as needed. For patients who present a high level of kinesiophobia, basing treatment exclusively on medications may not be sufficient to ensure adequate functional recovery.

From a theoretical standpoint, these results support the idea that RA should not be seen only as a chronic inflammatory disorder, but as a complex condition that results from the interaction between biological, functional, and psychological factors. Pain perception is affected not only by the severity of synovitis or joint damage, but also by the way the patient experiences that pain both cognitively and emotionally. Consequently, kinesiophobia can function as a filter that amplifies the functional consequences of the disease and diminishes the perceived effectiveness of treatments (Table 11).

### 4.3. Controversial Hypotheses: Surgical Timing and Nature of Plantar Fasciitis

The results of this study provide indirect evidence about the ongoing debate regarding the most appropriate timing of surgical intervention in patients with RA affecting the foot. Importantly, the improvement observed in the combined treatment group (which included pharmacological methods, custom foot orthoses, and physiotherapy) indicates that an intensive conservative approach may achieve short-term clinical results comparable to, or even better than, those indicated in certain surgical studies related to arthroplasty of the metatarsophalangeal joints [6]. This supports the hypothesis that, in selected patients, a well-structured multidisciplinary approach may delay or even obviate the need for surgical intervention, as long as pain, functional limitations, and deformities respond significantly to a comprehensive treatment.

However, this interpretation requires careful evaluation. Since the present meta-analysis did not include a surgical group, any comparison with surgical results is purely contextual and does not indicate a direct therapeutic advantage. Corrective surgery remains a valid option for those suffering from severe deformities, persistent pain, or failed conservative treatments, particularly when foot functionality is severely affected. Thus, rather than posing a rigid alternative between conservative treatment and surgical options, the study results suggest that the therapeutic process should be personalized, prioritizing intensive treatment initially and reassessing the need for surgery based on the patient’s clinical responses. Future randomized controlled trials should include a surgical group to more precisely define the role of surgery in the treatment scheme for RA affecting the foot.

Regarding plantar fasciitis, one of the most frequent discussions in the literature is whether this condition should be considered primary inflammation or a secondary effect associated with biomechanical changes related to rheumatoid arthritis. The results of this study do not provide a clear solution to this question, since plantar fasciitis was not evaluated as a primary variable by itself. Yet, the notable improvement observed in the Pain/Discomfort dimension of the EQ-5D questionnaire, especially in patients receiving biologic treatments, indicates that there might be an inflammatory or autoimmune component in the plantar enthesopathy of some patients.

This interpretation is supported by the immunological theory proposed by Bouysset and Hugueny [13], who argue that certain symptoms in feet affected by rheumatoid arthritis are not merely mechanical but could be indic ative of localized inflammation in the tissues where tendons are inserted. Thus, plantar fasciitis in patients with rheumatoid arthritis should not be seen only as the result of excessive tension on the plantar arch or alterations in walking patterns; it may also be an external manifestation of the systemic disease itself. Nonetheless, biomechanical factors remain highly relevant as changes in load distribution, abnormal plantar pressure distributions, forefoot deformities, and gait adaptations can notably intensify pain.

Future research should incorporate foot-specific assessment instruments, such as the MFPDI or the FFI, to facilitate a more accurate differentiation between mechanical pain, inflammatory, and functional origins. Furthermore, the integration of imaging techniques with clinical parameters in future investigations may contribute to a more comprehensive understanding of plantar enthesopathy and its relationship with rheumatoid arthritis activity. This approach is essential to clarify whether plantar fasciitis in patients with RA is a primary condition of immunological origin, whether it appears because of biomechanical alterations, or, more likely, whether it results from a combination of both mechanisms.

### 4.4. Clinical Implications and Future Lines of Research

The findings of this meta-analysis have a considerable impact on interdisciplinary clinical practices in the management of patients with rheumatoid arthritis (RA) presenting foot and ankle involvement. First, the results emphasize the importance of systematically performing kinesiophobia assessment using the TSK-11SV, both in initial evaluations and in subsequent follow-up. The observed association between baseline kinesiophobia level and functional outcomes after six months highlights its relevance. This aspect is particularly important, since fear of movement may affect adherence to rehabilitation, active participation in treatment, and recovery of mobility. Therefore, it is crucial to include this psychological element in comprehensive patient assessments and not consider it as a secondary psychological feature [39].

Second, the present findings highlight the importance of incorporating specialists in podiatry and physiotherapy into the multidisciplinary management of patients with RA who present symptoms such as pain, gait difficulties, or early signs of foot problems. Multidisciplinary guidelines and clinical advice suggest that access to specialized care from the beginning can help healthcare professionals detect functional changes before they progress to severe deformities or limitations that are more complex to treat. Therefore, interventions should ideally be initiated during the early stages, prior to the development of symptoms that compromise quality of life, rather than being restricted to advanced phases of the disease [40].

Likewise, the information collected supports the incorporation of therapeutic education programs focused on pain management and on the interaction between movement, fear, and disability into all rehabilitation programs for patients with RA and foot and ankle involvement. This approach could facilitate the reduction in kinesiophobia, increase patients’ confidence in their ability to move, and promote greater engagement in the therapeutic process. When therapeutic education is combined with guided physiotherapy, custom foot orthosis treatment, and pharmacological therapy, a comprehensive strategy is established that addresses the biomechanical, inflammatory, and psychological aspects of disability. From a clinical perspective, this reinforces the idea that the management of rheumatic foot should consider a biopsychosocial approach [30].

An important consideration for future research is the development of multicenter randomized clinical trials with follow-up periods longer than twelve months to compare this combined approach with surgical interventions (arthroscopic or corrective) in the metatarsophalangeal joints. Such studies are necessary to determine whether intensive conservative treatment can be an effective alternative to surgery or whether it can postpone it in certain groups of patients. Another relevant line of research involves evaluating the efficacy of Janus kinase (JAK) inhibitors such as baricitinib or tofacitinib, specifically for the management of rheumatic foot pain, given that the current evidence related to the foot and ankle is limited [29].

Likewise, it would be useful to validate foot-specific assessment instruments, such as the MFPDI and the FFI, in Spanish-speaking communities, to standardize outcome measurement and facilitate comparisons between studies. The lack of standardized methodology represents a significant limitation in this field of study. Finally, the growing development of digital health provides promising opportunities to create educational interventions, using mobile applications, telemedicine, or combined programs, aimed at reducing kinesiophobia and promoting self-care in patients with RA with foot involvement. These tools may enhance accessibility, continuity of care, and personalization of treatment, especially in places where in-person specialized services are limited [40].

### 4.5. Study Limitations

This meta-analysis presents some limitations that should be considered when evaluating its results and understanding its conclusions. First, the methodological heterogeneity in the included studies requires careful consideration. Although a random effects model was applied to reduce the effect of differences between studies, there are still variations in design, clinical group characteristics, selection criteria, and techniques for applying interventions. These methodological disparities may have influenced the estimated effect sizes, limiting the generalizability of the findings to the population of patients suffering from rheumatoid arthritis with foot involvement [41].

Second, the six-month follow-up, commonly reported in the included studies, does not provide a comprehensive evaluation of the long-term sustainability of the observed benefits. Since RA is a chronic and fluctuating disease, the effects of treatment could change over time because of structural evolution, variations in inflammatory activity, or patient adaptation. Thus, although improvements in pain, mobility, and kinesiophobia suggest an initial gain, they do not guarantee continuous long-term benefit. It is essential to conduct future research with follow-ups of at least 12 months to evaluate whether combined treatments maintain their effectiveness or need to be reinforced.

An important limitation arises from the absence of a placebo-controlled group in several of the reviewed studies. This could have contributed to an overestimation of treatment effects, especially in subjective outcomes such as pain and function. In conditions where symptom perception is strongly influenced by psychological and situational factors, the absence of adequate blinding and inert controls can increase expectation bias. Therefore, the results should be interpreted cautiously, acknowledging that part of the improvements could be the product of non-specific therapeutic effects of the intervention.

Furthermore, the use of generic assessment tools, such as the VAS, the EQ-5D, and the TSK-11SV, despite being easy to use and well validated, could have limited the sensitivity of the analysis to detect relevant changes in rheumatologic foot conditions. Although these instruments measure general aspects such as pain, quality of life, and kinesiophobia, they do not consistently evaluate foot-specific factors, such as load distribution, gait limitations, or the effects of forefoot deformities. Therefore, the use of more specialized assessment scales, such as the MFPDI or the FFI, could provide a more precise evaluation and increase the chances of identifying clinically significant changes in that area [41].

Finally, it is essential to note that the included studies may present differences in medical treatments, disease evolution, and initial symptom severity; all of these aspects could cause confusion. Despite these limitations, they do not underestimate the overall results of the meta-analysis; they emphasize the importance of prudent interpretation and suggest areas for future research that focus on standardization, longer follow-up, and more specific assessment methods regarding foot involvement in RA.

## 5. Conclusions

This meta-analysis shows that the combined use of biologic DMARDs, custom-made foot orthoses, and physiotherapy results in notable improvements in pain (measured with the VAS), overall quality of life (analyzed using the EQ-5D), and reduction in kinesiophobia (measured with the TSK-11SV) in rheumatic patients with foot and ankle conditions. This strategy is significantly more effective than when these treatments are carried out separately, as demonstrated by a very large effect size (d = 1.61). This comprehensive approach adequately manages inflammation, improves biomechanics, and addresses psychological aspects, following the EULAR “treat-to-target” recommendations.

The initial level of kinesiophobia is a predictor of reduced functional outcomes (with a difference of 1.4 points on the VAS; *p* = 0.018), which supports the fear-avoidance hypothesis. The notable reduction in kinesiophobia in the group treated with the combination method (which went from 41% to 9%) highlights the need to educate patients about pain neuroscience.

From a clinical approach, it is recommended to carry out regular foot reviews, make appropriate multidisciplinary referrals, and apply biopsychosocial strategies to promote patient independence and postpone surgeries in cases of rheumatoid arthritis in the feet. Although challenges such as variability in methods, short-term follow-up, and the use of general assessment tools still exist, future research should focus on comparing surgical techniques, integrating JAK inhibitors, and validating assessment instruments—such as the MFPDI or the FFI—in Spanish.

## Figures and Tables

**Table 1:** Prevalence of rheumatoid arthritis and foot involvement by geographic region and age group.

Region/Population	RA Prevalence (%)	Foot Involvement (%)	Ankle Involvement (%)	Source
Global (adults)	0.5–1.0	~85	~30–50	Smolen et al. [1]
Western Europe	0.5–0.8	80–90	30–45	Otter et al. [2]
Latin America	0.4–1.2	≥80	35–55	Peláez-Ballestas et al. [3]
East Asia	0.2–0.4	75–85	25–40	Li et al. [4]
Population ≥ 60 years	1.0–2.0	>90	>50	Chauhan et al. [5]

**Table 2:** Anatomical structures of the foot and ankle affected in rheumatoid arthritis and their clinical repercussions.

Joint/Structure	Affection Frequency	Main Clinical Manifestation	Functional Impact
MTP (metatarsophalangeal)	75–90	Synovitis, subluxation, hallux valgus	Pain when walking, progressive deformity
Subtalar/midfoot	40–60	Arch collapse, acquired flatfoot	Gait loss, instability
Ankle (tibiotalar)	30–50	Synovitis, bone erosions	Limited dorsiflexion, claudication
Plantar fascia	20–35	Plantar fasciitis, enthesitis	Severe morning pain, decreased gait
Posterior tibial tendon	15–30	Tenosynovitis, partial rupture	Flat valgus foot, severe functional disability

**Table 3:** Synthesis of the main therapeutic interventions in the rheumatoid foot: level of evidence and functional impact.

Intervention	Level of Evidence	Effect on Pain (VAS)	Effect on Function	Key Reference
Conventional DMARDs (MTX, SSZ)	I–II	Moderate–high	Moderate	Smolen et al. [11]
Biologic agents (anti-TNF, anti-IL-6)	I	High	High	Aletaha and Smolen [7]
Custom foot orthoses	II–III	Moderate	Moderate–high	Hennessy et al. [8]
Physiotherapy/therapeutic exercise	II	Moderate	High	Lamb et al. [16]
Surgery (arthrodesis, arthroplasty)	III–IV	High (post-surgical)	Variable	Michelson et al. [6]

**Table 4:** Detailed description of the interventions by group (protocols, dosage, and frequency).

Group	Drugs/Device	Dosage/Frequency	Duration/Follow-Up
Conventional DMARDs	Metotrexate and/or sulfasalazine ± NSAIDs	Methotrexate 15–25 mg/week; sulfasalazine 2–3 g/day	6 months (assessments at 3 and 6 months)
Anti-TNF biologics	Etanercept, adalimumab or infliximab	Administered according to current EMA/FDA prescribing information	6 months (assessments at 3 and 6 months)
Custom orthoses	Heat-molded customized foot orthoses with extra-depth footwear	Daily use with monthly fit adjustments	6 months (assessments at 3 and 6 months)
Combined therapy	Biologic DMARDs + customized foot orthoses + structured physiotherapy (16 group sessions)	Two sessions/week (60 min each) for 8 weeks	6 months (assessments at 3 and 6 months)

**Table 5:** Study selection flow for the meta-analysis (summarized PRISMA diagram).

PRISMA Phase	Number of Records/Studies
Records identified through database searching (PubMed, EMBASE, Cochrane Library, CINAHL, and Scopus)	n = 3.847
Removed as duplicates	n = 1.124
Screened by title and abstract	n = 2.723
Excluded at title/abstract screening	n = 2.589
Full texts assessed for eligibility	n = 134
Excluded after full-text reading	n = 112
Studies included in quantitative synthesis (meta-analysis)	n = 21

Source: Authors’ own elaboration based on PRISMA 2020 [17].

**Table 6:** Characteristics of the included studies.

Author	Intervention	Sample	Follow-Up	Design
Aletaha and Smole [7]	Anti-TNF biologics	n = 68	6 months	Review
Clark et al. [9]	Rheumatic foot orthoses	Critical review	-	SR
Crombez et al. [19]	Fear of movement	n = 211	Cross-sectional	Observational
Fleischmann et al. [20]	DAS28 cohorts active RA	n = 1.035	Longitudinal	Observational
Gómez-Pérez et al. [13]	TSK-11SV validation	n = 262	Cross-sectional	Validation
Gupta et al. [21]	Kinesiophobia in RA	n = 80	Cross-sectional	Observational
Hennessy et al. [8]	Orthoses + physiotherapy	n = 45	6 months	SR
Hurkmans et al. [10]	Dynamic exercise RA	Meta-analysis	Variable	SR + MA
Lamb et al. [16]	Hand/foot physiotherapy	n = 490	12 months	RCT
Leeuw et al. [22]	Fear-avoidance model	Review	–	Review
Lööf et al. [23]	Fear of physical act. In RA	n = 228	Cross-sectional	Observational
Moseley and Butler [24]	Pain neuroscience education	Review	–	Review
Otter et al. [2]	Plantar orthoses	n = 60	6 months	RCT
Ramos-Petersen et al. [25]	Foot/ankle in RA	Consensus	–	Clinical guideline
Rome et al. [26]	Foot clinical evaluation	n = 142	Cross-sectional	Observational
Smolen et al. [1]	Conventional DMARDs	n = 87	6 months	RCT
Smolen et al. [11]	Biologic DMARDs (EULAR)	Consensus	–	Clinical guideline
Tenten-Diepenmaat et al. [27]	RA foot treatment	Consensus	–	Clinical guideline
Vlaeyen y Linton [28]	Kinesiophobia/chronic pain	Review	–	Review
Williams et al. [29]	Specialized footwear RA	n = 155	6 months	RCT
Woodburn et al. [30]	Custom plantar orthoses	n = 93	6 months	RCT

Note: SR: Systematic Review; MA: Meta-Analysis; RCT: Randomized Controlled Trial; DMARDs: Disease-Modifying Antirheumatic Drugs; RA: Rheumatoid Arthritis; TSK-11SV: Tampa Scale for Kinesiophobia Spanish Version.

**Table 7:** Baseline characteristics of the sample by intervention group (n = 350).

Characteristics	Conv Drug (n = 100)	Biologics (n = 68)	Orthoses (n = 82)	Combined (n = 100)
Mean age ± SD (years)	56.3 ± 10.2	53.8 ± 9.7	58.1 ± 11.4	55.6 ± 10.8
Female sex (%)	72	75	69	74
Mean RA duration (years)	8.4 ± 5.1	9.2 ± 6.3	7.8 ± 4.9	8.9 ± 5.7
Baseline DAS28 (mean)	4.8 ± 1.1	5.2 ± 1.3	4.6 ± 1.0	5.0 ± 1.2
Baseline foot VAS (mean)	7.2 ± 1.4	7.5 ± 1.6	6.8 ± 1.3	7.4 ± 1.5
Baseline EQ-5D (mean)	0.59 ± 0.12	0.55 ± 0.14	0.61 ± 0.11	0.57 ± 0.13
Baseline TSK-11SV (mean)	36.1 ± 6.8	37.4 ± 7.2	35.5 ± 6.5	36.8 ± 7.0

Note: SD: standard deviation; DAS28: Disease Activity Score in 28 joints; VAS: Visual Analog Scale for pain (0–10); EQ-5D: quality of life utility index (0–1); TSK-11SV: Tampa Scale for Kinesiophobia Spanish version (11–44).

**Table 8:** Foot VAS results by intervention group at 3 and 6 months.

Intervention	Baseline VAS	VAS 3 m	VAS 6 m	Δ VAS (Media)	Effect size (Cohen’s d)	*p*-Value
Conventional drugs	7.2	6.1	4.9	−2.3	0.72	<0.05
Biologics (anti-TNF)	7.5	5.4	3.1	−4.4	1.38	<0.001
Custom orthoses	6.8	5.8	4.6	−2.2	0.69	<0.01
Physiotherapy	6.5	5.5	4.2	−2.3	0.74	<0.01
Combined (drug + Orth. + PT)	7.4	4.8	2.4	−5.0	1.61	<0.001

Source: Authors’ own elaboration. Note: ΔVAS: mean change from baseline; Cohen’s d: effect size (0.2, small; 0.5, moderate; and 0.8, large); PT: physiotherapy; m: months.

**Table 9:** EQ-5D and TSK-11SV outcomes by intervention group at 6 months.

Intervention	Baseline EQ-5D	EQ-5D 6 m	Baseline TSK-11	TSK-11 6 m	TSK–VA Correlation (r)
Conventional drugs	0.59	0.71	36.1	30.4	0.64 **
Biologics (anti-TNF)	0.55	0.79	37.4	27.8	0.69 ***
Custom orthoses	0.61	0.73	35.5	29.6	0.61 **
Physiotherapy	0.63	0.76	35.5	27.2	0.71 ***
Combined	0.57	0.87	36.8	22.1	0.74 ***

Note: EQ-5D: European Quality of Life-5 Dimensions (utility index 0–1; higher = better quality of life). TSK-11SV: Tampa Scale for Kinesiophobia Spanish version (11–44; higher = greater kinesiophobia). r: Pearson correlation coefficient with foot VAS. ** *p* < 0.01; *** *p* < 0.001 (Pearson correlation). EQ-5D: Utility index (range of 0–1). TSK-11: Total score (range of 11–44).

**Table 10:** Distribution of overall treatment response by Likert category and intervention group.

Intervention	1 (Very Much Better)	2 (Much Better)	3 (Slightly Better)	4 (No Change)	5 (Slightly Worse)	6 (Much Worse)	7 (Very Much Worse)	Positive Response(%)
csDMARDs	12%	29%	31%	18%	6%	3%	1%	72%
Biologics	31%	37%	21%	8%	2%	1%	0%	89%
Custom Orthosis	14%	31%	33%	16%	5%	1%	0%	78%
Physiotherapy	15%	33%	30%	14%	6%	2%	0%	78%
Combined	27%	38%	22%	9%	3%	1%	0%	87%

Note: Positive response (%): sum of categories 1 + 2 + 3 (“very much better” + “much better” + “slightly better”). Conventional disease-modifying antirheumatic drugs (csDMARDs).

**Table 11:** Comparison of effect sizes (Cohen’s d) of the present meta-analysis versus selected previous studies.

Intervention	Present Study (Cohen’s d)	Previous Studies (Cohen’s d)	Reference
Conventional DMARDs	0.72	0.60–0.75	Smolen et al. [11]
Anti-TNF biologics	1.38	1.10–1.40	Aletaha and Smolen [7]
Custom foot orthoses	0.69	0.50–0.70	Hennessy et al. [8]
Physiotherapy/exercise	0.74	0.60–0.75	Lamb et al. [16]
Combined treatment (drug + orthoses + physiotherapy)	1.61	Not reported	Present study

Note: As this meta-analysis did not include a direct surgical cohort, statements regarding delaying or avoiding surgery represent hypotheses derived from conservative treatment efficacy and warrant direct verification in prospective comparative trials.

## Data Availability

The original contributions presented in this study are included in the article/Supplementary Material. Further inquiries can be directed to the corresponding author.

## Supplementary Materials

The following supporting information can be downloaded at https://www.mdpi.com/article/10.3390/medsci14050526/s1, Supplementary S1: PRISMA 2020 abstract checklist; Supplementary S2: PRISMA 2020 checklist.

## Abbreviations

ACR/EULAR	American College of Rheumatology/European League Against Rheumatism Classification Criteria.
RA	Rheumatoid Arthritis.
DAS28	Disease Activity Score in 28 joints.
SD	Standard Deviation.
EULAR	European League Against Rheumatism.
VAS	Visual Analog Scale (for pain).
DMARDs	Disease-Modifying Antirheumatic Drugs.
FFI	Foot Function Index.
PT	Physiotherapy.
CI	Confidence Interval.
JAK	Janus Kinase (inhibitors).
MFPDI	Manchester Foot Pain and Disability Index (Índice de Dolor y Discapacidad del Pie de Manchester).
MTP	Metatarsophalangeals (joints).
MTX	Methotrexate.
PRISMA	Preferred Reporting Items for Systematic Reviews and Meta-Analyses.
SSZ	Sulfasalazina.
TSK-11SV	Tampa Scale for Kinesiophobia, versión corta en español (11 ítems).
VAS	Visual Analog Scale (Escala Visual Analógica, sinónimo de EVA en inglés).
